# Three years of banning neonicotinoid insecticides based on sub‐lethal effects: can we expect to see effects on bees?

**DOI:** 10.1002/ps.4583

**Published:** 2017-05-04

**Authors:** Tjeerd Blacquière, Jozef JM van der Steen

**Affiliations:** ^1^ Wageningen Plant ResearchWageningen University & ResearchWageningenthe Netherlands

**Keywords:** pollinator declines, exposure and dose, honey bees, neonicotinoids, pesticide ban

## Abstract

The 2013 EU ban of three neonicotinoids used in seed coating of pollinator attractive crops was put in place because of concern about declining wild pollinator populations and numbers of honeybee colonies. It was also concluded that there is an urgent need for good field data to fill knowledge gaps. In the meantime such data have been generated. Based on recent literature we question the existence of recent pollinator declines and their possible link with the use of neonicotinoids. Because of temporal non‐coincidence we conclude that declines of wild pollinators and of honeybees are not likely caused by neonicotinoids. Even if bee decline does occur and if there is a causal relationship with the use of neonicotinoids, we argue that it is not possible on such short term to evaluate the effects of the 2013 ban. In order to supply future debate with realistic (field) data and to discourage extrapolating the effects of studies using overdoses that are not of environmental relevance, we propose – in addition to field studies performed by the chemical industry – to use the ‘semi‐field worst case’ treated artificial diet studies approach to free flying colonies in the field. This kind of study may provide realistic estimates for risk and be useful to study realistic interactions with non‐pesticide stressors. © 2017 The Authors. Pest Management Science published by John Wiley & Sons Ltd on behalf of Society of Chemical Industry.

## 1 INTRODUCTION

The 2013 ban^1^ of three neonicotinoids used as seed treatments in pollinator attractive crops was based on reports and concern about honey bee colony declines as well as concern about declines of wild bees distribution and abundance. In addition, many reports on the sub‐lethal effects of neonicotinoids on bees, fuelled the concerns about the role of neonicotinoids in declines. The ban was also intended to gain extra time to fill some identified knowledge gaps. Here we first ask the question (1) whether such declines indeed occur, both for wild bees and honey bees, and when and where. If such declines are occurring, the next question (2) would be whether the use of neonicotinoids is a likely cause, both based on (non‐) coincidence (was the decline preceded by the neonicotinoid exposure?) and (expected) exposure levels. Not only have declines of honey bee stocks been reported,^2^ but in many regions of the world the beekeepers have been confronted with sudden losses of colonies.^3^ We ask (3) whether declines of honey bee stocks are driven by these colony losses (which in turn might have been enhanced by exposure to neonicotinoids). Therefore we ask (4) if neonicotinoids are indeed likely to be involved in the reported losses of honey bee colonies, which was a reason and motivation for the ban, then (5) are we to expect to see positive effects on bees of the ban of the three neonicotinoids in seed coatings, and if so, how fast? Finally, (6) what kind of additional data would we need then to better evaluate the risks of applying neonicotinoids as seed treatment in agriculture?

## 2 DECLINES

Declines of wild pollinator species and dependent plant species have been reported by Biesmeijer *et al*.,^4^ by comparing field observation data from the UK and Netherlands pre‐ and post‐1980. More detailed studies later showed strong declines of species richness, especially during the early post‐war decades (1950–1970), fewer from 1970–1990, and hardly any between 1990 and 2009.^5^ The principal driver of species richness declines of wild bees has been the intensification of agriculture resulting in a loss of habitat and pollen‐providing plants.^6^ The trend of declining pollinators seems to have slowed down in the latest two decades (1990–2010),^5^ the period that saw the introduction and increasing use of neonicotinoids.^7^ Moreover the pollinator species common in agricultural settings, and possibly contributing most to crop pollination, which are probably also the species most exposed, did not show declining trends compared to the other species mostly found outside agricultural areas.^8^

For honeybees, worldwide declines have been frequently claimed to occur and were taken as a sign of an underlying problem to be solved. However, worldwide there is a steady increase in the number of (managed) honey bee colonies,^9^ accompanied by an equally steady increase in honey production. Moreover, although there was a decrease in the rate of growth of the honey bee stock in the early nineties, this was mainly caused by the sudden collapse of apiculture in the former socialistic republics in Eastern Europe (including Russia).^9^ It appears that in both the USA and Canada as well as in parts of Europe numbers of colonies have increased since 2000.^2^, ^10^, ^11^, ^12^, ^13^ Losses of honey bee colonies, especially over winter have been reported from many countries and regions,^14^ although not from the southern hemisphere.^3^

## 3 INVOLVEMENT OF NEONICOTINOIDS IN DECLINES OF COLONY NUMBERS

For honey bee declines of colony numbers a Hill's analysis,^15^ as well as a causal analysis plus weight of evidence approach^16^ concluded exposure to neonicotinoids to be an unlikely cause for local declines of honey bee colonies worldwide and in California for almond pollinating colonies. Also several reviews summarising/analysing the evidence drew attention to the many factors that might play their roles in driving the declines, besides pesticides.^10^, ^12^, ^17^, ^18^ Although the latter are foremost in public discussions and policy plans, this does not reflect the analyses presented, and Smith *et al*.^11^ argue that more attention needs to be given to other causes than pesticides, particularly pathogens and parasites.

## 4 ROLE OF COLONY LOSSES IN DECLINES OF STOCKS

Although (increased) colony losses could sometimes be linked to or correlated with specific drivers, the increased incidence of colony losses in Europe,^3^ as well as the appearance of the Varroa mite in the USA could not be linked to declining numbers of colonies. The reason for the absence of such link is that beekeepers can rather easily recover their stock by splitting surviving colonies in spring.^13^ Several socio‐economic drivers, including the honey market and demand for pollination, proved to be far more impacting on numbers of managed honey bee colonies.^10^, ^11^, ^13^, ^17^

## 5 ARE NEONICOTINOIDS INVOLVED IN HONEY BEE COLONY LOSSES?

Although no direct relationship between colony losses and the decline of numbers of honey bee colonies could be shown (see section 4), losses of a high number of colonies over winter are considered to be a problem in itself for beekeepers, and have stimulated the foundation of the Coloss network.^14^ So could honey bee colony losses experienced by beekeepers be attributed to the use of neonicotinoids? Smith *et al*.^11^ point out that the evidence is not strong for the case. Even a very extensive 4‐year monitoring project set‐up with the intention to shed light on the possible factors involved in honey bee colony losses, and specifically focusing on residues of chemicals,^19^ was not able to show any relationship with these, but did show effects of infestation with the Varroa mite, some viruses and the age of the queen: actually all being part of the management choices of the beekeeper, a decisive factor often overlooked.^20^ Circumstantial evidence from a winter losses enquiry among a random sample of the Dutch beekeepers by telephone since 2013 (see section 6) also suggests that exposure to pesticides is not a likely explanation of differences between beekeepers in the numbers of colonies lost: it was found that 60–70% of the beekeepers did not lose any colonies, and beekeepers with and without losses were evenly distributed over the country,^21^ irrespective of agricultural intensity and likely neonicotinoid use.

## 6 WILL THE BAN READILY MITIGATE POLLINATOR DECLINES AND COLONY LOSSES?

Neonicotinoids may continue to be suspected in bee declines, though we feel the evidence for this is weak (see above, section 4). Even if they were involved in bee declines, would it be possible to observe effects from the ban on neonicotinoids in Europe in so short a time, being aware of the natural fluctuations in population dynamics of wild bees, as well as the fluctuations in winter losses of honey bee colonies? For wild bees most extinctions took place in the 1950s onwards. The fluctuations in the data only permitted to compare and analyse – with some fidelity – three sets of two subsequent decades.^5^ What could we confidently conclude after only 2 to 3 years of banning of three neonicotinoids in seeds of pollinator attractive plants? Given the arguments in section 4 and section 5 we would not expect to see effects on honey bee colony numbers.

A judgement is equally difficult for honey bee colony *losses*. Although Coloss^14^ has put a lot of effort in yearly collection of winter mortality data combined with many additional data in many European countries (and more) through surveys, these data have not all been completely published. Moreover, in some cases changes have been made over the years in the questions asked, which may impair the comparability between years.^22^, ^23^, ^24^ Rather extensive data are available for the winter of 2012–2013 (2 years before the ban became effective) and 2015–2016 (2 years after the ban became effective). In Europe as a whole mean losses were higher in 2012–2013 than in 2015–2016, and this was similarly true for all individual countries. However, when comparing 2 full years before and 2 full years after the ban, losses in Europe were similar (12.6 before, 14.2 after).^14^ However, the data of 2014 and 2015 is to date only available in Coloss press releases^14^ as preliminary results. The challenge for the ‘Monitoring Working Group’ of Coloss (http://www.coloss.org/coreprojects/monitoring) is now to publish an overview of all colony loss data collected and to analyse these in relation to data on seed treated pollinator attractive crops. Doing this analysis we should keep in mind that the ban became effective in December 2013, and knowing that the seeds of oil seed rape (flowering of 2014) had been sown the summer before, the first sowings without neonicotinoid treatment in Europe would have been those of 2014, resulting in flowers with no residues in nectar and pollen in the spring of 2015, and resulting in the first non‐exposed honeybee colony cohort to overwinter during the 2015–2016 winter. For crops flowering in the same year of sowing (maize, soybean, sunflower, spring sown oil seed rape) 2014 would be the first year with no carry over of residues across successive years in nectar and pollen, and spring of 2015 the first moment to see possible effects on winter losses. Moreover, all other possible drivers should be analysed too, specificity being one of the criteria in the Hill's epidemiological analysis.^15^ For instance Switanek *et al*.^25^ showed by analysing data of 6 years in Austria a connection between colony losses and temperature as well as precipitation in the preceding summer.

## 7 NEED FOR REALISTIC SUB‐LETHAL DOSING STUDIES IN THE FIELD

Most of the supporting evidence for the ban came from (laboratory‐) studies on sub‐lethal effects of neonicotinoids, of which the observed effects were often extrapolated to the real world. However, all concentrations not killing the insects are by definition sub‐lethal but only concentrations between field rate and zero are possibly relevant. We previously reviewed effects and levels of dosing/exposure: apart from guttation^26^ and abrasion at sowing,^27^ the expected exposures (through pollen and nectar) were all below hazardous levels.^7^ This was best illustrated in a paper by Walters^28^ showing that published studies finding no effects on bees used concentrations from 1/100th to 100 times the found concentrations in nectar or pollen, whilst the studies showing effects ranged from around the field concentrations observed up to 10 000 (one even 1 000 000) times that concentration. The majority of studies reporting effects used concentrations and doses much higher than typical field exposures. Since then many papers have been published stressing the use of field realistic concentrations. However, supplying a big volume (for instance by a long duration of dosing) with a field realistic concentration yet results in a high dose,^28^ and in many studies use of ‘field realistic concentrations’ actually results in ‘worst case dosing’ or even worse than that.^29^

The relevance of a certain concentration of a pesticide in nectar or pollen depends strongly on the exposure of bees to the contaminated nectar or pollen. For example, there could be a dilution effect whereby, if there are several attractive crops available to bees to forage on, the residue load per crop could diminish. Garbuzov *et al*.^30^ decoded the waggle dances of honey bees together with analysis of the incoming pollen loads to the hive and showed that the share of oil seed rape foraging (nectar and pollen) was only minor (between 4 and 14% of the pollen), despite oil seed rape being seemingly the most attractive crop present (with an area coverage of ∼3%). The relative amount of foraging on oil seed rape was far higher (62–83% of oil seed rape pollen in the harvested honey, 10% to >80% in pollen traps), for oil seed cultures in a recent monitoring study in Germany.^31^

Although the sensitivity of bees to most chemicals (but with exceptions) does not differ strongly between species (including the ‘sentinel’ honey bee),^32^, ^33^, ^34^ it is obvious that life history traits of the bees do strongly determine ways and the level and duration of exposure and the consequences of these in the field.^35^ Therefore field data for one species cannot be extrapolated to another species, not even if at the same location.

Because there is much confusion about effects and concentrations and to which extent observed effects relate to the real situation in the field, as well as about the status of much of the evidence, two restatements have been written after ample discussion.^36^, ^37^ With the aim to clarify and to deduce exposures of bees and colonies from reported concentrations in nectar and pollen, several calculations have been made to relate different scale studies. It appears that already an updated restatement might be needed in view of the tremendous supply of recent research papers in the field.

To underpin the decision on a possible continuation or a relief of the ban more good field data and realistic exposure studies have been asked by EFSA (European Food Safety Authority). Such studies are very elaborate and expensive and most probably would be only possible when carried out by the chemical industry. Fortunately in recent years a series of such elaborate studies have been performed and published.^31^, ^38^, ^39^, ^40^

A compromise between laboratory plus semi‐field studies and the real field (monitor) studies may be ‘treated artificial feeding studies’, in which free foraging honey bee or bumblebee colonies are fed ‘in‐colony’ field‐realistic concentrations of the pesticide in sugar syrup.^41^, ^42^ The dosing and the duration of these studies capture a full range of exposure concentrations, including concentrations and doses that exceed realistic worst cases. This kind of study is very useful to explore the thresholds above which risk of hazard starts, or below which the risks of hazard are acceptably low. In addition, with treated artificial diet feeding studies it is possible to better gauge the impacts of interactions with other stressors (f.i. lack of forage, Varroa mite infestation, see Dively *et al*.^42^ and Blanken *et al*.^43^). We discuss here the relevant treated artificial diet studies with honey bees regarding the dosage of sugar and imidacloprid per colony and per bee.^41– 46^ Wu‐Smart and Spivak^44^ fed small honey bee colonies of three sizes (1500, 3000 and 7000 bees per colony) with five concentrations (0 (=control), 10, 20, 50, 100 ng g^−1^) of imidacloprid in sugar syrup (50% w/v), during 21 days. They observed significant negative effects of imidacloprid exposure on the egg laying rate of the queens, on the hygienic behaviour (removal of capped brood cells that had been freeze killed) and on the share of empty cells in the capped brood pattern of the colonies. Only the empty cells in the brood pattern showed a dose related response, the egg laying rate of the queen was inhibited by about 50% compared to the control, at all concentrations. This indicates that at 10 ng g^−1^ already the full scale negative effect on egg laying had been reached, and increasing the concentration even up to ten fold did not add to the effect. In risk assessment research this experiment would have been considered a ‘range finding test’, which urges to carry out the ‘real experiment’ with a series of concentrations between 0 and 10 ng g^−1^, the range which also includes the field‐realistic concentrations, which average 2 ng g^−1^ in nectar.^36^

All colonies apart from those in the study by Meikle *et al*.^46^ were only fed a small part (one third to half) of the needed sugar, calculated as 19–36 mg of sugar per day per bee from Rortais *et al*.^47^ (Table 1 Column 4). This mimics a worst case scenario for foraging, with up to half of all forager bees of a colony foraging on contaminated nectar. In addition, in the study by Wu‐Smart and Spivak^44^ the concentration used to mimic contaminated nectar is at least four times the so far to be expected concentration in nectar. The study by Meikle *et al*.^46^ is quite different, as it simulates a high intake of (contaminated) nectar during a strong honey flow: indeed the quantity of sugar supplied per day per bee is more than six times the needed quantity of 19–36 mg, and the colonies did strongly increase in weight during the dosing period of 6 weeks.

**Table 1:** Sugar feeding and imidacloprid (imi) dosing in six published studies,^41^, ^42^, ^43^, ^44^, ^45^, ^46^ calculated per colony and per average bee

1. Study	2. Sugar per col per day (g)	3. No. bees	4. Sugar (mg) per bee per day	5. Imi (ng g^−1^ syrup or patty)	6. Imi (µg) per colony	7. Days dosed	8. Imi (ng) per bee life or dose period	9. Imi (ng) per bee per day	10. Effect bee/col
**(A) Dosing studies**									
Faucon *et al*.^41^	237	?15 000	15.8	0.5	7.8	34	0.428	0.015	ND/−
Dively *et al*.^42^	Patty 46	15 000	—	5	16.6–25.2	84	0.36–0.56	0.013–0.020	ND/−
Dively *et al*.^42^	Patty 46	15 000	—	20	63.7–126	84	1.46–2.80	0.052–0.100	ND/+
Van der Steen *et al*.^45^	57	5 000	11.5	5	51	84	10.2	0.12	ND/−
Blanken *et al*.^43^	58	5 000	11.7	6	58.5	84	3.90	0.14	−/−
Faucon et al.^41^	237	?15 000	15.8	5	78	34	4.28	0.153	ND/−
Meikle *et al*.^46^	380	20 000	190	5	160	42	5.3	0.19	ND/−
Dively *et al*.^42^	Patty 46	15 000	—	100	323–630	84	7.28–14	0.260–0.500	ND/+
Dively *et al*.^42^	Patty 400 g wk^−1^ * and sugar 1 kg wk^−1^	14 000	10	100	240	42	11.4	0.41	ND/+
Dively *et al*.^42^	Patty 400 g wk^−1^ and sugar 1 kg wk^−1^ **	14 000	10	20	240	42	11.4	0.41	ND/−
Wu‐Smart and Spivak^44^	100	7 000	14	10	84	21	12	0.58	+/−
Meikle *et al*.^46^	380	20 000	190	20	638	42	21.3	0.76	ND/−
Wu‐Smart and Spivak^44^	100	7 000	14	20	168	21	24	1.16	+/−
Wu‐Smart and Spivak^44^	100	7 000	14	50	420	21	60	2.90	+/+
Meikle *et al*.^46^	380	20 000	190	100	3192	42	106	3.80	ND/+
Wu‐Smart and Spivak^44^	100	7 000	14	100	854	21	122	5.80	+/+
**(B) Monitoring studies**		**N bees**			**Clo /colony (µg)**	**Days**	**Clo/bee (ng)**	**Clo/bee/day (ng)**	**Effect** **bee/col**
Cutler and Scott‐Dupree^38^		11 000			<25.0	21	<2.3	<0.11	ND/−
Cutler *et al*.^39^		11 000			<35.4	14	<3.2	<0.23	ND/−
Rolke *et al*.^31^, ^48^		20 000			43.4	28	2.17	0.08	ND/−

* Patty was spiked with imidacloprid 100 ng g^−1^;

** sugar syrup was spiked with imidacloprid 20 ng g^−1^.

(A) The daily amount of sugar supplied per bee (Column 4) was derived from the dosing per colony (C 2) and the estimated average number of adult bees per colony (C 3). For comparison an expected sugar consumption per average bee of 19–36 mg sugar day^−1^ was calculated from Rortais *et al*.,^47^ assuming 30% of bees foraging, and 50% of these for pollen (a relatively high share of pollen foragers because of the daily sugar feed for free), and calculating a mean energy consumption of in hive bees of 18 mg per bee per day. The dosed imidacloprid per colony (C 6) was derived from the used concentration in the syrup or patty (C 5) and the quantities supplied during the dosing period. The daily dose per average bee (C 9) was derived from the daily dose given to the colonies (C 6), divided by the number of adult bees present in the colonies (C 3). To estimate a (chronic) total dose per bee over its life time (C 8) the daily dose (C 9) was multiplied by 28 (supposed life time of a worker bee), or by the duration of the dosing if less than 28 days. Column 10 roughly summarizes reported effects at bee and colony levels: +: effect; −: no effect; ND: not determined. Studies and parts of studies have been arranged in order of increasing dose per bee (Column 9).

(B) Exposure of colonies to neonicotinoid residues (Column 6, µg clothianidin per colony), calculated from average honey yield and concentration of residues in honey; average number of bees per colony (Column 3) and the calculated amount of residue per average bee (C 8, ng per bee) and daily dose (C 9, ng per bee per day) from studies by Cutler and Scott‐Dupree,^38^ Cutler *et al*.^39^ and Rolke *et al*.^31^, ^48^

Remarkably only those studies using the higher doses (>0.20 ng per average bee per day in patties, >0.58 ng per average bee per day in syrup) show in some cases sub‐lethal effects (Table 1, C 10), whilst this is not the case when using field realistic or lower concentrations. This is not different from the overview given in Walters.^28^ But even more than comparability among dosage studies it is relevant how these dosages relate to real exposure which bees and bee colonies experience when foraging on a seed treated flowering crop. Therefore we also calculated the exposure on colony level and individual bee level for the available (large) field monitoring studies.^31^, ^38^, ^39^, ^48^ Table 1B shows that the exposures are lower per colony (Column 6) as well as per bee (Column 9) than most of the used doses in the treated artificial feeding studies. Nevertheless such treated artificial feeding studies approach the ‘natural’ exposure of colonies, although it still needs to be stressed that the real exposure of the monitoring studies for the major part ended up in the 25–40 kg honey per colony. This honey may be harvested by the beekeeper, and thus not expose the bees, or if it is not harvested it would expose the bees later and during a prolonged period when the bees start to consume the honey stock. In that case the exposure would be shared by a much higher number of bees than only the standing population during the honey flow and it will be dispersed over more time, resulting in lower daily doses. Meikle *et al*.^46^ showed that even rather high and stable residue levels did not provoke significant hazards in that scenario.

Bees receiving >0.58 ng per day^44^ accumulate >12 ng in a dosing time of 21 days, >16 ng in an average lifetime of 28 days. This dose is three to four times the acute oral 48 h LD_50_ of ∼4 ng per bee. Despite receiving several times the 48 h LD_50_ during a lifetime often no damage was observed. This indicates that bees must have been able to eliminate or detoxify the chemical. This was indeed demonstrated in a chronic feeding laboratory study,^49^ which showed that individual honeybees could detoxify 2.2 ng per 24 h, i.e. more than half the acute oral 48 h LD_50_. This also implicates that we should trust environmentally realistic *ad libitum* dosing over the laboratory acute dose (= single meal) paradigm. Moreover, the dose daily detoxified is at least ten times the dose per average bee in the three monitoring studies listed in Table 1B.

## 8 CONCLUSIONS

In this perspectives paper we ask whether improvements by the 2013 EU ban of neonicotinoids as seed treatments on the situation of pollinators could be observed and expected. However we concluded:

1. That wild pollinator declines did not increase during the neonicotinoid era.
2. The same was concluded for declines of honeybee colonies, and observed declines could be linked to other drivers than pesticides.
3. Honeybee colony losses, which did increase since 2000, were associated more with pests and parasites as well as with beekeeping practices, than with the use of neonicotinoids.
4. Moreover, although they pose a problem for beekeeping in a great part of the world, the losses of colonies of honey bees are not a likely driver for declining honey bee stocks.
5. We conclude that it will not likely be possible to see effects of the 2013 ban on so short notice (2 years only now) because of huge year to year variation in colony loss rates and because of the involvement of many other factors, and because colony losses data are collected inconsistently over the years.
6. However, the respite offered by the ban can be used to add needed data: extensive field monitor data (to be provided by the chemical industry) and in addition independent research data that provide sensitivity analyses of realistic doses or exposures. Treated artificial diet studies with free flying colonies provide an excellent tool to sense the limits of acceptable exposures of pesticides in the real world. It is, however, not enough to treat with ‘Field Realistic’ concentrations because it is not concentration but the dose that makes the poison.^29^
7. By comparison of field monitor data with effects on honey bee colonies in treated artificial diet studies we conclude that negative effects on honey bees of correct use of neonicotinoid seed treatments are unlikely to occur.
